# Risk Factors and Clinical Outcomes of Invasive Fungal Infections in Patients with Severe COVID-19: A Case–Control Study

**DOI:** 10.3390/pathogens14101064

**Published:** 2025-10-21

**Authors:** Nosheen Nasir, Syed Abbas Moazzam Kazmi, Joveria Farooqi, Muhammad Irfan, Kauser Jabeen

**Affiliations:** 1 Department of Medicine, Aga Khan University, Karachi 74800, Pakistan; nosheen.nasir@aku.edu (N.N.); muhammad.irfan@aku.edu (M.I.); 2 Medical College, Aga Khan University, Karachi 74800, Pakistan; syed.abbas24@alumni.aku.edu; 3 Department of Pathology and Laboratory Medicine, Aga Khan University, Karachi 74800, Pakistan; joveria.farooqi@aku.edu

**Keywords:** severe COVID-19, COVID-19-associated invasive fungal infections, invasive fungal infection (IFIs), candidiasis, aspergillosis, ICU mortality, invasive mechanical ventilation

## Abstract

Background: Invasive fungal infections (IFIs) in patients with COVID-19 contribute to significant morbidity and mortality, with reported incidence between 5% and 26.7%. COVID-19-associated pulmonary aspergillosis (CAPA), candidiasis (CAC), mucormycosis (CAM), and Pneumocystis jirovecii pneumonia (PJP) are the most common IFIs in this population. Methodology: We conducted a case–control study in the ratio of 1:2 between March 2020 and April 2022 using institutional COVID-19 registry data. The cases were severe COVID-19 patients with IFIs, and the controls were severe COVID-19 patients without IFIs. Multivariate logistic regression was used to identify independent risk factors, with adjusted odds ratios (aOR) and 95% confidence intervals (CIs). The outcomes for the study were to assess the clinical outcomes, i.e., in-hospital mortality and length of hospitalization in a subgroup of severe COVID-19 patients who had IFIs. A *p*-value < 0.05 was considered significant. Results: Among 5368 COVID-19 patients admitted to hospital during the study period, 1333 had a severe infection. Of these, 158/1333 (11.8%) met the criteria for IFIs, with a median age of 65 years and 71% male predominance. Diabetes (53.8%) and hypertension (57.6%) were the most common comorbid conditions. Acute respiratory distress syndrome (ARDS) developed in 55% of patients. Overall mortality was 48%. For the case control analysis, 119 patients with IFIs were selected as cases and 240 patients without IFIs were selected as controls. On univariate analysis ARDS was significantly associated with IFIs (OR: 1.91; 95% CI: 1.23–2.99, *p*-value = 0.004). Patients with IFIs had higher odds of being on hemodialysis compared to those without IFIs (OR: 2.17; 95% CI: 1.18–3.99; *p*-value = 0.013). Mechanical ventilation was found to be independently associated with IFIs in multivariate logistic regression analysis (OR: 2.5, 95% CI: 1.58–3.96, *p*-value < 0.001). The odds for in-hospital death in patients with IFIs were 2.19 (95% CI: 1.35–3.56; *p*-value < 0.001) compared to patients without IFIs. The median hospital stay for patients with IFIs was markedly longer (14 days) compared to 8 days in patients without IFIs. Conclusions: IFIs significantly worsened outcomes in severe COVID-19 patients, leading to increased mortality and prolonged hospital stays. Mechanical ventilation was an independent risk factor for IFIs.

## 1. Introduction

Invasive fungal infections (IFIs) are a major cause of morbidity and mortality in critically ill COVID-19 patients, with reported incidence between 5% and 26.7% [1]. The most common IFIs in COVID-19 patients are COVID-19 associated pulmonary aspergillosis (CAPA), COVID-19 associated mucormycosis (CAM), COVID-19 associated candidemia (CAC), and Pneumocystis jirovecii pneumonia (PJP). Further, cryptococcosis, fusariosis, and histoplasmosis are implicated as fungal infections in COVID-19 patients as well [2]. Risk factors for IFIs in this population include immunosuppressed states, such as malignancy. Other risk factors include lung disease, uncontrolled diabetes, and corticosteroid or immunomodulator use. Leukopenia and prolonged mechanical ventilation are also reported risk factors [3,4]. Additionally, one study highlighted the fact that most patients with CAPA were male and had a history of diabetes mellitus or chronic obstructive pulmonary disease [5].

The risk factors for IFIs have been variably reported from different parts of the world, likely influenced by the environment and local epidemiology. Pakistan was amongst the first few countries to report CAPA [6]. During the mid-2021 surge of COVID-19 caused by the highly transmissible Delta (B.1.617.2) variant, there was a noticeable rise in CAM reported from South Asia, including Pakistan [7,8,9].

Hence, it was imperative to determine the risk factors for IFI in hospitalized patients with severe COVID-19 for early recognition and management. Our study aimed to determine the risk factors for IFIs in patients with severe COVID-19 at a tertiary care center in Karachi, Pakistan. We restricted our study population to patients with severe COVID-19, as IFIs have been predominantly reported in this subgroup, particularly those requiring intensive respiratory support or immunomodulatory therapy. Including only severe cases in both the case and control groups minimized confounding due to disease severity and allowed for a more accurate assessment of risk factors specific to IFIs within a clinically relevant population.

## 2. Materials and Methods

This study was conducted at the Aga Khan University Hospital, Karachi, Pakistan. We conducted a case–control study in the ratio of 1:2 between March 2020 and April 2022 utilizing data captured in the hospital’s COVID-19 Registry. Cases were defined as all hospitalized patients with severe COVID-19 as per WHO criteria (defined as the presence of clinical signs of pneumonia plus respiratory rate > 30 breaths/min or severe respiratory distress, or SpO_2_ < 90% on room air) [10] confirmed with either an antigen test or PCR and identified to have an IFI. The controls were patients with severe COVID-19 who did not have IFIs during hospitalization. The outcomes for the case control study were the assessment of the clinical outcomes, i.e., in-hospital mortality and length of hospitalization in the subgroup of severe COVID-19 patients who had IFIs. Patients with CAPA were diagnosed using ECMM/ISHAM criteria modified to include tracheal aspirate culture and/or Galactomannan Index > 4.5 in the possible CAPA category. CAC was defined by isolation of *Candida* species from blood cultures. CAM was defined as updated EORTC/MSG criteria with inclusion of COVID-19 as a host factor. PJP was defined by consistent clinical, radiological, PCR positivity and 1,3-beta-D-glucan positivity [11,12,13,14].

Patients were eligible for inclusion as cases if they were aged 18 years or older, had laboratory-confirmed SARS-CoV-2 infection (via RT-PCR or antigen testing), met the World Health Organization (WHO) criteria for severe or critical COVID-19, and had a proven or probable IFI during the course of their illness. All cases were hospitalized during the same period as the control group to minimize temporal bias. Controls were selected from patients who also had severe or critical COVID-19 based on WHO definitions, but without any clinical, radiologic, or microbiologic evidence of IFIs during hospitalization, and whose hospital stays overlapped with those of the cases to control for seasonal and temporal variation. Patients were excluded if they had mild or moderate COVID-19, a pre-existing or chronic fungal infection prior to their COVID-19 diagnosis, incomplete medical records relevant to fungal diagnostics, or antifungal therapy.

We collected data on key pre-existing comorbid conditions known to influence the risk and outcomes of IFIs and severe COVID-19. The comorbidities assessed included diabetes mellitus, hypertension, coronary artery disease, chronic liver disease, chronic kidney disease, malignancies (solid and hematologic), chronic obstructive pulmonary disease, asthma, autoimmune diseases, cerebrovascular accident, and HIV infection. Diagnoses were extracted from patients’ electronic medical records and clinical documentation, based on physician-assigned diagnoses made prior to or during the index hospitalization. Where applicable, standard clinical definitions were used, such as HbA1c ≥ 6.5% or ongoing use of glucose-lowering therapy for diabetes, and eGFR < 60 mL/min/1.73 m^2^ for chronic kidney disease. Malignancies were included if there was a history of active disease or ongoing treatment. Autoimmune diseases included systemic lupus erythematosus, rheumatoid arthritis, and similar conditions requiring immunosuppressive therapy. HIV status was confirmed through documented serology or existing diagnosis in the medical record.

We restricted the study population to severe COVID-19 cases—both in the COVID-IFI group and the control group—based on clinical relevance and methodological rigor. IFIs have been predominantly observed in patients with severe or critical illness, particularly those requiring high-flow oxygen, mechanical ventilation, or immunosuppressive therapies such as corticosteroids or IL-6 inhibitors. Including mild or moderate cases in the control group would have introduced selection bias and confounded the associations, given their inherently lower baseline risk of IFIs. Restricting the analysis to severe cases ensured that both groups shared a similar risk profile with respect to immune compromise, antimicrobial and steroid exposure, and ICU-level care. This approach enhanced internal validity and improved the ability to identify risk factors specific to IFIs, rather than differences driven by disease severity alone. It also aligns with clinical utility, as findings are most applicable to the population where screening and antifungal stewardship are most critical.

**Figure 1:**
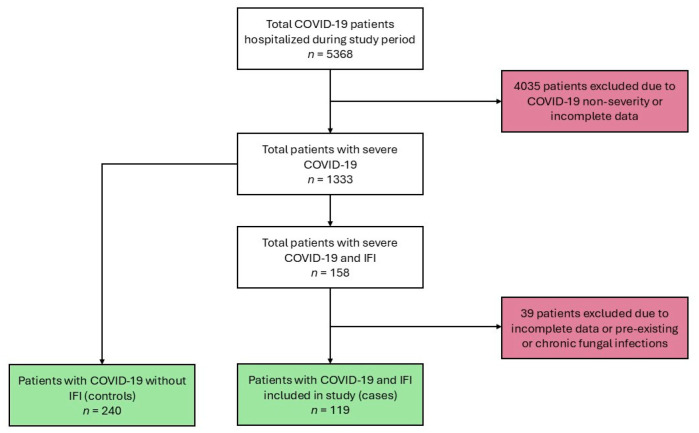
Flow chart showing cases and controls inclusion in the study.

In this case–control study, we identified a total of 5368 patients who were hospitalized with COVID-19 during the study period. From this cohort, 1333 patients with severe COVID-19 had complete clinical data records and were eligible for analysis. Of these 1333 patients, 158 (11.8%) met the criteria for IFIs. For the case control analysis, 119 patients with IFI were selected as cases. Controls (*n* = 240) were randomly selected from the remaining patients within the same cohort (Figure 1).

To identify factors independently associated with the development of IFIs among patients with severe COVID-19, univariate and multivariable logistic regression analyses were conducted. Continuous variables such as age and length of hospital stay were summarized using medians and interquartile ranges (IQRs), while categorical variables such as sex and comorbidities were reported as frequencies and percentages. Pearson’s Chi-square or Fisher’s exact test were used to compare categorical variables, as appropriate.

For multivariable logistic regression, variables with a *p*-value < 0.10 in univariate analysis, along with clinically relevant factors (e.g., corticosteroid use, ICU admission, mechanical ventilation), were included in the final model. Results were reported as adjusted odds ratios (aORs) with 95% confidence intervals (CIs). Multicollinearity was assessed using variance inflation factors (VIFs), and model fit was evaluated using the Hosmer–Lemeshow goodness-of-fit test. A *p*-value < 0.05 was considered statistically significant. All analyses were performed using Stata statistical software (version 17.0; StataCorp, College Station, TX, USA).

The study was reviewed and approved by the Ethics Review Committee of the Aga Khan University, Karachi, Pakistan. Due to the retrospective nature of data collection, the requirement of informed consent was waived as per institutional policies.

## 3. Results

### 3.1. Clinical Characteristics of COVID-19 Patients with IFIs

**Figure 2:**
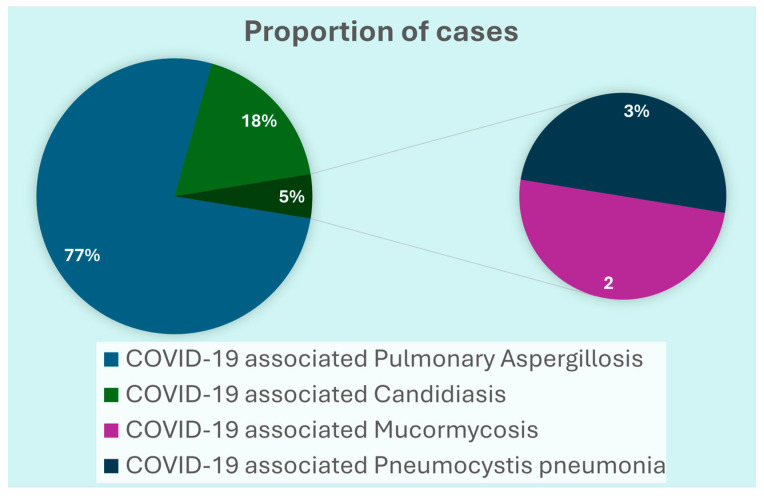
Distribution of invasive fungal infections over the study period.

Of the 1333 severe COVID-19 patients, 158 (11.8%) fulfilled the criteria for IFIs. The median age of the patients with IFIs was 65 (Range: 20–94) years and the majority *n* = 112 (71%) were male. The notable comorbidities were diabetes *n* = 85 (53.8%) and hypertension *n* = 91 (57.6%). Forty-eight (30.4%) patients had undergone surgical procedures during hospitalization. More than half of the patients *n* = 87 (55%) had acute respiratory distress syndrome (ARDS) and *n* = 68 (43%) required invasive mechanical ventilation indicating severe to critical COVID-19. Among the IFIs, the majority had CAPA [*n* = 119 (75%)], followed by CAC [*n* = 28 (18%)], CAM [*n*= 4 (2.5%)], and PJP [*n* = 7 (4.4%)] (Figure 2). *Aspergillus flavus* [*n* = 62 (39.2%)] and *Aspergillus niger* [*n* = 31 (19.6%)] were the most common fungal organisms. Amongst CAC, *Candida albicans* [*n* = 10 (35.7%)] was the most common, followed by *C. parapsilosis* [*n* = 5 (17.8%)], *C. glabrata* [*n* = 5 (17.8%)], *C. tropicalis* [*n* = 4 (14.3%)], *C. auris* [*n* = 2 (10.7%)] and *C. rugosa* [*n* = 1 (3.6%)]. Two-thirds of patients were found to have concurrent bacterial growth. Almost all of the patients received systemic steroids during hospitalization [*n* = 154 (97.5%)].

There was no statistically significant association between any of the comorbid conditions, including diabetes, and IFIs (*p*-value = 0.788). Antifungals were administered to *n* = 72 (45.6%) patients, with voriconazole as the primary treatment modality in *n* = 68 (43%), though it was not found to be protective of mortality in this cohort (*p*-value = 0.133). Overall mortality was *n* = 76 (48%) and median length of hospitalization was 13 days (Range 1–62). Among the IFIs, the highest mortality was observed with CAM, with 3 out of 4 (75%) deaths.

### 3.2. Comparison of Severe COVID-19 Patients with IFIs with Those Without IFIs

Out of 158 patients with IFIs, 119 with severe COVID-19 were compared to 240 patients without IFIs. The median age of patients with IFIs was 64 years, compared to 60 years in patients without IFIs. Most of the patients were male in both groups. There were no significant differences between the two groups in terms of comorbid conditions and treatment received (Table 1). However, patients with IFIs compared to those without IFIs had 1.91 times the odds of having ARDS (95% CI: 1.23–2.99, *p*-value = 0.004). Moreover, patients with IFIs had higher odds of being on hemodialysis compared to those without IFIs (Table 1). In multivariate regression analysis, mechanical ventilation was found to be independently associated with IFIs in severe COVID-19 (aOR: 2.5; 95% CI: 1.58–3.96; *p*-value < 0.001) after checking for appropriate interactions and confounding by other co-variates.

**Figure 3:**
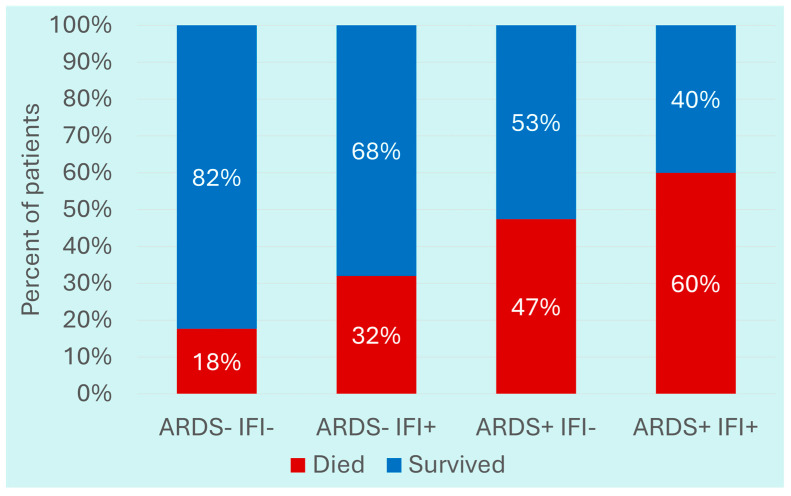
Association between ARDS and mortality in patients with COVID-19 and invasive fungal infections.

Among the outcomes, the odds for in-hospital death in patients with IFIs were 2.19 (95% CI: 1.35–3.56; *p*-value < 0.001) times higher than patients without IFIs. Furthermore, 60% of COVID-19 patients with IFIs who had ARDS during hospitalization died compared to 32% of those who did not have ARDS (*p*-value < 0.001) (Figure 3).

The median hospital stay for patients with IFIs was markedly longer (14 days) compared to 8 days in patients without IFIs (Table 1).

## 4. Discussion

The intersection of COVID-19 and IFIs has emerged as a critical area of concern, particularly among critically ill patients, and studies highlight that IFIs, such as CAPA, CAM, and CAC significantly exacerbate disease severity and mortality. Our study found that patients with IFIs were at greater odds of requiring mechanical ventilation. Furthermore, the clinical outcomes were worse, with increased odds for in-hospital mortality and longer length of hospitalization in patients with IFIs. The current study confirmed the findings of previous studies and underscored the critical importance of timely diagnosis and management of IFIs to improve clinical outcomes in severe COVID-19 cases.

Variable diagnostic criteria across studies led to a widely reported incidence of CAPA in patients with COVID-19 acute respiratory failure requiring invasive ventilation (median 20.1%; range 1.6–38%). The adoption of stricter European Confederation of Medical Mycology/International Society for Human and Animal Mycoses (ECMM/ISHAM) criteria in 2020 significantly reduced the reported CAPA prevalence to about 10% among invasively ventilated patients [15], with a systematic review even reporting it as 3.7% [16]. CAC had an overall prevalence of 2.4% [16], while CAM was reported in 0.27% of hospitalized patients with COVID-19 in India [15]. A systematic review by Hussain et al. reported the pooled prevalence of CAM as 0.7% [17]. COVID-19-associated pneumocystis pneumonia (PCP) was found to have a prevalence of 1.7% [18], although there is a paucity of data on this. Similarly, a nested case–control study in Mexico by Roman-Montes et al. reported an overall IFIs prevalence of 9.3% in ICU patients, with CAPA accounting for 60% (47/78) of the patients with IFIs, followed by invasive candidiasis in 32% (25/78), pulmonary cryptococcosis in 5% (3/78), and pulmonary mucormycosis in 5% (3/78) [19].

In the study by Roman-Montes et al., cases of IFIs were at increased odds of having prolonged duration of invasive mechanical ventilation (>21 days) (*p*-value = 0.02) [19]. A multicenter study in France also emphasized that patients with IFIs were ventilated on average 5.4 days longer than those without (OR: 1.01; 95% CI 1.00–1.02; *p*-value = 0.0098) [20]. A systematic review by Chong et al. also concluded that CAPA patients had a prolonged duration of invasive mechanical ventilation, ranging from 13 to 20 days [21]. Among patients with COVID-19 undergoing invasive mechanical ventilation, studies have reported a high incidence of invasive aspergillosis in up to 30% of intubated patients [22]. In another systematic review, it was reported that 29.4% of patients with CAM were mechanically ventilated [23]. In these patients, the mortality rate was 78.5%, in comparison to 29.3% in patients that did not require mechanical ventilation (*p*-value < 0.001) [23].

COVID-19 patients with ARDS, prolonged ICU or ventilator dependence, and those receiving high-dose corticosteroids, immunomodulators, interleukin inhibitors, or broad-spectrum antibiotics, face an increased risk of developing mucosal candidiasis, CAPA, CAM, PJP, and candidemia [2,24].

A single-center study concluded that hospitalized patients with IFIs were admitted for a longer duration (63 days) and had a higher mortality rate (23%) compared with COVID-19 patients who did not develop IFIs [25]. Those who did not develop IFIs were hospitalized for 24 days and had a mortality of 7% [25]. Another single-center study reported that patients with IFIs had longer ICU stays (26 vs. 13 days, *p*-value < 0.001) and a higher risk of in-hospital death (69% vs. 36%, *p*-value < 0.001) [26].

IFIs in COVID-19 patients dramatically increased mortality. A 2024 U.S. study of 1934 COVID-19 patients with IFI concluded that the incidence of IFIs was found to be 2.8% among intubated patients. In this study, *Aspergillus* and *Candida* species were the most common pathogens, similar to what our study highlighted. This study reported a 90-day all-cause mortality of 50.9% in IFI cases versus 36.8% in non-IFI controls (*p*-value < 0.0001) [27]. Angioinvasive CAPA with a positive galactomannan is associated with >80% mortality [15]. Patients with persistent viral shedding, particularly those who are immunocompromised, are also at an elevated risk of developing invasive aspergillosis and face higher mortality rates [28].

In a study from Pakistan, 39% of patients with COVID-19 were found to have either *Aspergillus* species colonization or CAPA. The fatality rate was 44% [6]. Another observational study in Pakistan found that 10 out of 280 patients with COVID-19 requiring mechanical ventilation were found to have CAM. Five patients died, while two left the hospital against medical advice in critical condition and were presumed to have died [29]. Furthermore, a case–control study highlighted the fact that patients with CAPA had a significantly greater percentage of complications and a longer length of stay (*p*-value  < 0.001). Patients with CAPA also had a higher mortality rate (48%) compared to those without CAPA (13.5%) [OR = 6.36 (95% CI 3.6–11)] [30].

Candidemia is a commonly reported secondary infection among critically ill patients with extended hospital stays. A narrative review reported that case reports and observational studies involving 401 cases of IFIs have shown high crude mortality rates of 56.1% and 74.8%, respectively [2]. PCP has a mortality rate of up to 52.9% in non-HIV patients [31], while a systematic review by Amstutz et al. reported a mortality rate of 30.9% in patients with COVID-19-associated PCP [32].

A systematic review highlighted that risk factors such as poorly controlled diabetes mellitus, structural lung disease, and immunosuppressive therapies contribute to the susceptibility of COVID-19 patients to IFIs [3]. Other studies continue to highlight diabetes and steroid administration as significant risk factors [33,34]. In fact, in a study from Pakistan, diabetes was the most common comorbidity in CAPA and CAM [6,35]. Hypertension, obesity, chronic kidney disease, chronic obstructive pulmonary disease, central venous catheters, hemodialysis, and prolonged hospital stay (or ICU admission) also serve as risk factors for IFIs, particularly disseminated candidiasis [36,37,38,39].

Although our study suggests that hemodialysis and prolonged hospital stays show significant associations with IFIs, diabetes and hypertension were not significantly associated with IFIs. We attributed this to the fact that these comorbidities are highly prevalent in the general population, regardless of whether or not the patients have severe COVID-19. Chronic kidney disease and lung disease also did not show significant associations, possibly due to the small sample size in these respective categories.

In a study conducted in Malta, 9.5% of patients presenting with COVID-19 ARDS were found to have confirmed and probable IFIs [40]. A British study concluded that, among COVID-19 patients who presented with ARDS, the prevalence of invasive yeast infections and CAPA was 12.6% vs. 14.1%, respectively [12]. Our univariate analysis showed that patients with COVID-19 who developed ARDS had 1.91 times higher odds of having an IFI compared to those without ARDS. This initial association suggests that ARDS may contribute to the development of IFIs, potentially due to factors such as prolonged mechanical ventilation. However, after adjusting for other variables in multivariate regression, ARDS was no longer a significant independent predictor, indicating that its effect on IFI risk may be largely explained by other factors, particularly mechanical ventilation. We believe that the loss of significance was due to the fact that both groups of patients had severe COVID-19, making ARDS a defining feature in both. This reduced the contrast needed to detect a significant independent association with IFIs.

A 2024 multicenter study of 559 mechanically ventilated COVID-19 patients found that each additional day of corticosteroid use increased the risk of fungal infections requiring ≥5 days of antifungal therapy [41]. Concurrent use of interleukin-6 inhibitors like tocilizumab also acts as a risk factor for developing fungal infections, particularly CAPA [15,36]. In our study, almost every patient received corticosteroids, and there was no significant difference between the two populations. Notably, studies continue to highlight the use of corticosteroids as a double-edged sword. Although systemic corticosteroids may reduce mortality in severe COVID-19 patients, they may also predispose them to IFIs [42,43].

These studies thus underscore the need to control comorbidities, and, when suspecting IFIs, investigate further with fungal biomarkers and cultures in those patients who are clinically deteriorating despite optimal medical treatment, as well as possibly considering empirical anti-fungal treatment if suspicion remains high.

The use of voriconazole was not associated with a reduction in mortality among patients with IFIs in our study. Studies have reported variable responses to treatment and most have been small observational studies [4,44]. The most commonly utilized antifungal was voriconazole, with trends towards better outcomes, although these outcomes did not reach statistical significance [22]. However, a recent study has shown no decrease in mortality with antifungals in COVID-19 patients with IFIs, similar to our cohort, although they had 81% patients on antifungal therapy [45]. Our study is limited by small sample size and may have been underpowered to detect meaningful differences in outcome due to antifungals. Furthermore, our data lacks information on time to initiation of antifungals, which can also impact treatment outcomes.

Our study highlights the risk factors and outcomes of IFIs in COVID-19 patients from a low/middle-income country (LMIC) to provide valuable insights.

Our findings align with prior studies reporting elevated rates of IFIs, particularly aspergillosis and candidemia, among critically ill COVID-19 patients. However, unlike reports from India and other South Asian countries during the same period, we did not observe high numbers of CAM cases in our study. This discrepancy may be attributed to differences in population-level risk factors, such as lower baseline prevalence of uncontrolled diabetes. For example, a meta-analysis found that diabetes mellitus was reported in approximately 74.5% of CAM cases, reflecting trends seen in countries like Mexico and Iran. In comparison, European studies from Italy, France, and Greece have shown significantly lower rates of diabetes among CAM patients, ranging from 18% to 29%, indicating that other underlying conditions—particularly hematological malignancies—may contribute more substantially to CAM risk in those populations [17]. In comparison, in our study, 47.1% of cases of IFI had diabetes, significantly less than the number reported in literature. The lower rates of CAM in our study could also be due to differences in corticosteroid dosing practices, or earlier implementation of antifungal stewardship protocols in our setting.

This study offers a unique contribution to the growing literature on IFIs, particularly from an LMIC context where data remain scarce. Unlike many previous studies, we focused exclusively on patients with severe or critical COVID-19, thereby ensuring a high-risk and clinically relevant comparison group while minimizing confounding by disease severity. By applying standardized international diagnostic criteria and controlling for temporal bias through overlapping hospital stays between cases and controls, our methodology enhances both internal validity and comparability with global data. However, the study was single-center, limiting its generalizability to tertiary care centers in LMICs. Another limitation was missing data for IFI cases. Additionally, its retrospective nature makes it susceptible to selection bias and residual confounding as the data did not measure prior antifungal prophylaxis or antifungal treatment. Despite these limitations, our findings highlight the need for early recognition and management of IFIs to improve patient outcomes.

## 5. Conclusions

IFIs significantly worsen outcomes in severe COVID-19, leading to increased mortality and prolonged hospital stays. Mechanical ventilation was independently associated with IFIs, highlighting the role of critical illness and intensive care interventions in their development. The lack of association with traditional risk factors such as diabetes and hypertension may be due to the uniformly severe nature of COVID-19 in both groups. These findings emphasize the need for heightened clinical vigilance, to mitigate the impact of IFIs in critically ill COVID-19 patients.

## Figures and Tables

**Table 1:** Comparison of patients with invasive fungal infections with those without invasive fungal infections in hospitalized patients with severe COVID-19.

Variables	IFIs (*n* = 119)	No IFIs (*n* = 240)	Unadjusted OR (95% CI)	*p*-Value
Median Age (IQR) years	64 (54–70)	60 (52–69)	1.01 (0.99–1.03)	0.317
Sex n (%)				
Male	85 (71.4)	159 (66.3)	1.27 (0.79–2.06)	0.375
Female	34 (28.6)	81 (33.6)	Ref	
Comorbidity n (%)				
DM	56 (47.1)	134 (55.8)	0.70 (0.45–4.13)	0.118
HTN	65 (54.6)	141 (58.7)	0.85 (0.54–1.32)	0.457
IHD	8 (6.72)	16 (6.70)	1.01 (0.42–2.43)	0.984
CKD	19 (19.0)	9 (3.8)	1.25 (0.54–1.32)	0.422
Chronic Lung Disease	13 (10.9)	20 (8.3)	1.30 (0.65–2.82)	0.425
Malignancy	6 (5.0)	10 (4.2)	1.22 (0.43–3.44)	0.706
Medications n (%)				
Tocilizumab	35 (29.4)	53 (22.1)	1.47 (0.89–2.42)	0.130
Steroids	117 (98.3)	233 (97.1)	1.76 (0.36–8.59)	0.486
Antibiotic given n (%)	118 (99.2)	229 (95.4)	5.67 (0.72–44.4)	0.099
Invasive mechanical ventilation n (%)	56 (47.1)	63 (26.2)	2.48 (1.57–3.96)	<0.001
Hemodialysis n (%)	24 (20.2)	25 (10.4)	2.17 (1.18–3.99)	0.013
ARDS n (%)	62 (52.1)	87 (36.3)	1.91 (1.23–2.99)	0.004
In-hospital Mortality n (%)	49 (46.7)	61 (28.5)	2.19 (1.35–3.56)	0.001
Length of hospital stay (median (IQR))	14 (8–20)	8 (5–13)	1.06 (1.04–1.09)	<0.001

Abbreviations: IFIs: Invasive Fungal Infection; DM: Diabetes Mellitus; HTN: Hypertension; IHD: Ischemic Heart Disease; CKD: Chronic Kidney Disease; ARDS: Acute Respiratory Distress Syndrome; IQR: Interquartile range.

## Data Availability

Data is contained within the article.

## Abbreviations

The following abbreviations are used in this manuscript:

IFI	invasive fungal infection
CAPA	COVID-19-associated pulmonary aspergillosis
CAC	COVID-19-associated candidiasis
CAM	COVID-19-associated mucormycosis
PJP	Pneumocystis jirovecii pneumonia
ARDS	acute respiratory distress syndrome
OR	odds ratio
